# Molecular Characterization of Gastric Epithelial Cells Using Flow Cytometry

**DOI:** 10.3390/ijms19041096

**Published:** 2018-04-06

**Authors:** Kevin A. Bockerstett, Chun Fung Wong, Sherri Koehm, Eric L. Ford, Richard J. DiPaolo

**Affiliations:** Department of Molecular Microbiology and Immunology, Saint Louis University School of Medicine, Saint Louis, MO 63104, USA; kevin.bockerstett@slu.edu (K.A.B.); johnny.wong@health.slu.edu (C.F.W.); sherri.koehm@health.slu.edu (S.K.); eric.ford@health.slu.edu (E.L.F.)

**Keywords:** flow cytometry, gastric epithelium, autoimmune gastritis, atrophic gastritis

## Abstract

The ability to analyze individual epithelial cells in the gastric mucosa would provide important insight into gastric disease, including chronic gastritis and progression to gastric cancer. However, the successful isolation of viable gastric epithelial cells (parietal cells, neck cells, chief cells, and foveolar cells) from gastric glands has been limited due to difficulties in tissue processing. Furthermore, analysis and interpretation of gastric epithelial cell flow cytometry data has been difficult due to the varying sizes and light scatter properties of the different epithelial cells, high levels of autofluorescence, and poor cell viability. These studies were designed to develop a reliable method for isolating viable single cells from the corpus of stomachs and to optimize analyses examining epithelial cells from healthy and diseased stomach tissue by flow cytometry. We performed a two stage enzymatic digestion in which collagenase released individual gastric glands from the stromal tissue of the corpus, followed by a Dispase II digestion that dispersed these glands into greater than 1 × 10^6^ viable single cells per gastric corpus. Single cell suspensions were comprised of all major cell lineages found in the normal gastric glands. A method describing light scatter, size exclusion, doublet discrimination, viability staining, and fluorescently-conjugated antibodies and lectins was used to analyze individual epithelial cells and immune cells. This technique was capable of identifying parietal cells and revealed that gastric epithelial cells in the chronically inflamed mucosa significantly upregulated major histocompatibility complexes (MHC) I and II but not CD80 or CD86, which are costimulatory molecules involved in T cell activation. These studies describe a method for isolating viable single cells and a detailed description of flow cytometric analysis of cells from healthy and diseased stomachs. These studies begin to identify effects of chronic inflammation on individual gastric epithelial cells, a critical consideration for the study of gastric cancer.

## 1. Introduction

Chronic atrophic gastritis is a common complication after infection with *Helicobacter pylori* and in individuals that develop autoimmune gastritis [1]. Chronic atrophic gastritis is a major risk factor for developing gastric cancer, which is the third most common cause of cancer-related deaths worldwide [2,3]. The pathophysiology of gastric cancer development has been well studied in several mouse models using primarily histopathological microscopy techniques [4]. While these are the standard techniques to analyze progression of pathologic changes in gastric epithelial tissue, there are difficulties in obtaining organ-wide surveys of epithelial cells. Proper statistical analysis would require the counting of numerous cells in many different areas of tissue [5]. With respect to these technical difficulties, flow cytometric analysis is ideal for measuring protein expression on individual gastric epithelial cells.

Flow cytometry relies on the identification of proteins using antibodies conjugated to fluorochromes that, when excited by incident light, emit fluorescence at distinct wavelengths. This enables identification of cell populations based on the wavelength of fluorescence detected [6]. Flow cytometry provides an organ-wide survey of protein expression that can be used to differentiate cell types, identify surface receptors, assess production of secreted protein products, determine activation state of transcription factors, and many other applications [7,8,9,10]. Flow cytometry analysis is used sparingly in the analysis of freshly isolated gastric epithelial cells partly due to the difficulties in tissue processing and data interpretation of highly autofluorescent populations [5,11,12,13].

The goal of this study was to provide a comprehensive methodology for single cell analysis of the complex gastric gland that is composed of parietal, chief, foveolar, and mucous neck cell types. This necessitated isolating individual cells from gastric corpus glands, staining for surface molecules, and gating that allows for analysis of gastric epithelial cells by flow cytometry. Generation of single cell suspension from the stomachs of BALB/c mice was assessed morphologically using cytospin preparations of gastric epithelial cells at various stages of digestion. Staining for antibodies against epithelial cell adhesion molecule (EpCAM) and cluster of differentiation (CD)45 were used to differentiate epithelial cells and hematopoietically derived immune cells, respectively. Analysis of gastric epithelial cells from control mice and from mice that develop autoimmune chronic atrophic gastritis (TxA23) allowed for a comparison of cells in the fundic mucosa under normal conditions and conditions of inflammatory gastric preneoplasia [14,15]. After generating single cell suspensions from cohorts of BALB/c and TxA23 mice, we used flow cytometry to: (1) Identify gastric epithelial cells; (2) identify immune cells in the gastric mucosa of mice with chronic atrophic gastritis; (3) identify parietal cells; and (4) demonstrate that inflammation causes a significant increase in major histocompatibility complex (MHC) molecules on the surface of gastric epithelial cells. The ability to isolate cells from the corpus mucosa, identify immune and epithelial cell populations using lineage specific markers, and analyze inflammation-induced changes by flow cytometry significantly enhances our ability to study the effects of chronic gastritis on the gastric epithelium in this model of gastric preneoplasia and others.

## 2. Results

### 2.1. Enzymatic Digestion Results in a Single Cell Suspension Comprised of Major Gastric Epithelial Cell Lineages

**Figure 1:**
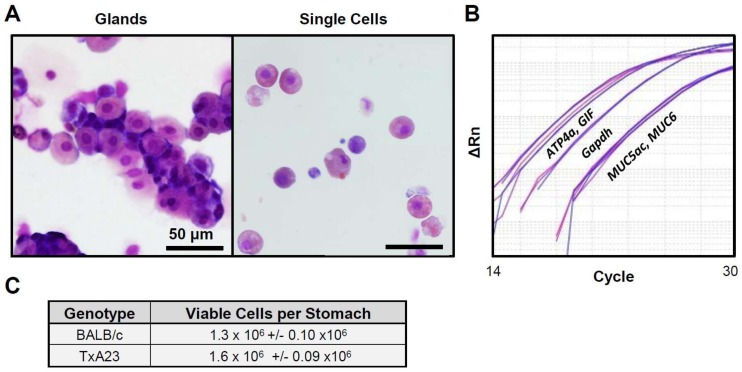
Isolating single cells from gastric corpus glands. (**A**) Representative cytospins of freshly isolated glands (left) and single cell suspensions generated from glands isolated from the corpus mucosa of stomach from mice (right). (**B**) qRT-PCR analysis of lineage markers for parietal cells (*Atp4a*), chief cells (*Gif*), surface mucous cells (*Muc5ac*), and mucous neck cells (*Muc6*). mRNA was isolated from gastric glands as seen in (**A**). (**C**) Table showing average viable cell number determined using Trypan blue exclusion from BALB/c and TxA23 mice. *n* = 3 mice per group.

As flow cytometric analysis requires single cell suspensions, we sought to optimize a protocol that yielded high numbers of gastric epithelial single cells. We isolated gastric glands using collagenase and observed normal glandular structure by light microscopy following cytospin preparation (Figure 1A, left). Glands were then further digested into single cells using Dispase II and Cytospin preparations, confirming a majority of single cells present in solution (Figure 1A, right). We next isolated RNA from these single cell suspensions and assessed the major gastric epithelial cell lineages present using quantitative RT-PCR probes against parietal cells (*Atp4a*), chief cells (*Gif*), surface mucous cells (*Muc5ac*), and mucous neck cells (*Muc6*). This analysis determined that all of these major lineage transcripts were present, particularly *Atp4a* and *Gif*, which had crossing threshold (CT) values lower than that of the control target *Gapdh* (Figure 1B)*.* Finally, we performed cell counting using Trypan blue exclusion and observed that these isolation methods yield on average over 1 × 10^6^ viable cells (Figure 1C). Therefore, this epithelial cell isolation method generates single cell suspensions that contain major cell populations present in the stomach.

### 2.2. Optimizing Flow Cytometric Analysis of Gastric Epithelial Cells

**Figure 2:**
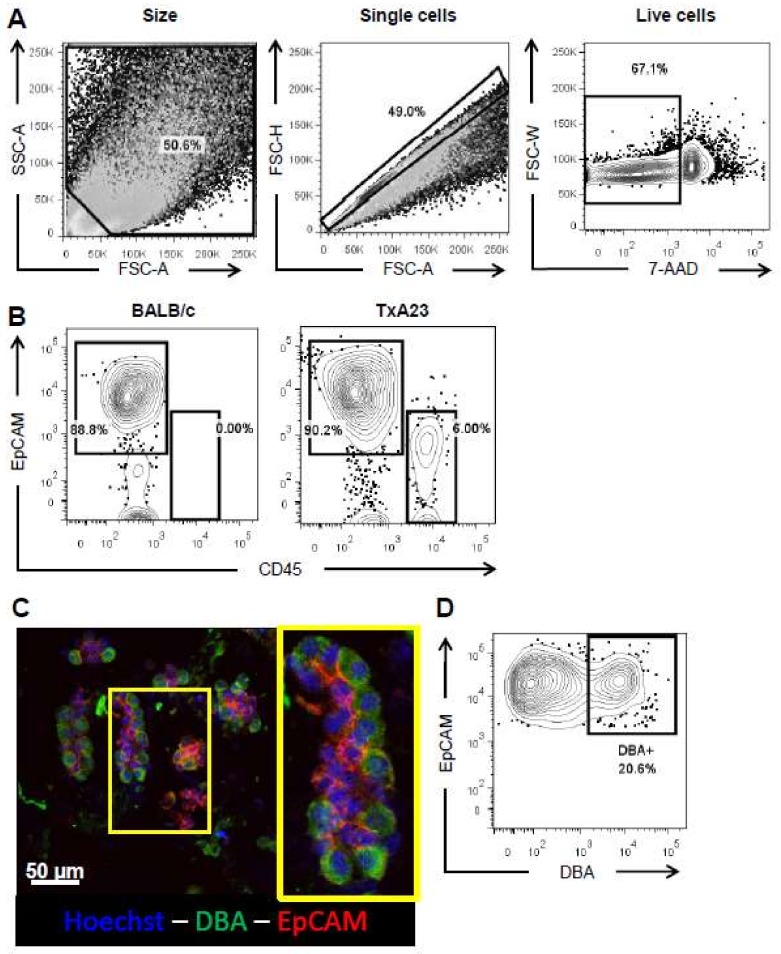
Gating strategy for analyzing gastric epithelial cells by flow cytometry. (**A**) A gate based of forward area and side scatter area is first established, these cells are then put through a forward scatter area and forward scatter height gate to identify single cells, finally, 7-AAD is used to separate live cells from dead/dying cells; (**B**) Representative flow plots of live single cells from healthy BALB/c and TxA23 mice stained with an epithelial cell marker (EpCAM) and an immune cell marker (CD45). Immune cells are undetectable in the gastric mucosa control mice, and present in TxA23 mice that have autoimmune gastritis; (**C**) Representative immunocytochemistry of glands isolated from a 2 month old BALB/c mouse and stained with hoechst (blue), parietal cell marker Dolichous bifluorous agglutinin (DBA, green), and anti-EpCAM (red) with a high magnification inset in yellow showing an individual gland; (**D**) A representative flow cytometry plot of live single cells from a BALB/c mouse stained with anti-EpCAM and DBA demonstrating a subset of EpCAM + DBA + parietal cells.

Gastric epithelial cells have proven to be difficult to analyze by flow cytometry, in part, due to the degree of cell-intrinsic autofluorescence and the tendency of epithelial cells to re-aggregate after isolation. To minimize autofluorescence and the improper analysis of cell doublets, we developed a strategy to: (1) Filter cells immediately before analysis, (2) exclude highly autofluorescent cell fragments according to size and internal complexity, (3) exclude aggregated cells according to the ratio of cell height to cell area, and (4) differentiate dead cells according to cell viability dye. The forward-scatter vs. side-scatter gate specifically excludes very small cell fragments and debris (Figure 2A, left). The single cell gate compares the height of a given event to its area, and events that fall along the central axis are single cells rather than aggregated multiplets (Figure 2A, center). Finally, dead cells are excluded using a viability dye, 7-aminoactinomycin D (AAD), that preferentially stains dying cells with porous membranes (Figure 2A, right). We then selected epithelial cells according to the expression of epithelial cell adhesion molecule (EpCAM/CD206). In the case of cells isolated from our mouse model of autoimmune gastritis, it is necessary to exclude infiltrating immune cells present in the sample using CD45 staining, a cellular protein expressed on all hematopoietically-derived cells (Figure 2B). These methods minimize autofluorescence and cell aggregation and provide a way to specifically analyze the effect of chronic inflammation on individual gastric epithelial cells while excluding hematopoietically derived immune cells. Finally, we wanted to use this methodology to identify a gastric epithelial cell lineage. *Dolichos biflorus* agglutinin (DBA) has been previously shown to be a parietal cell-specific staining reagent [16,17]. We performed immunocytochemistry on glands from BALB/c mice and stained them with Hoechst, DBA-fluorescein isothiocyanate (FITC), and anti-EpCAM antibodies and observed parietal cell specific staining (Figure 2C). We then adapted DBA staining to our flow cytometry analysis and observed a subset of EpCAM+cells that were also DBA+ (Figure 2D), demonstrating the ability to identify parietal cells using flow cytometry.

### 2.3. Gastric Epithelial Cells Upregulate MHC-I and MHC-II in Response to Chronic Inflammation

**Figure 3:**
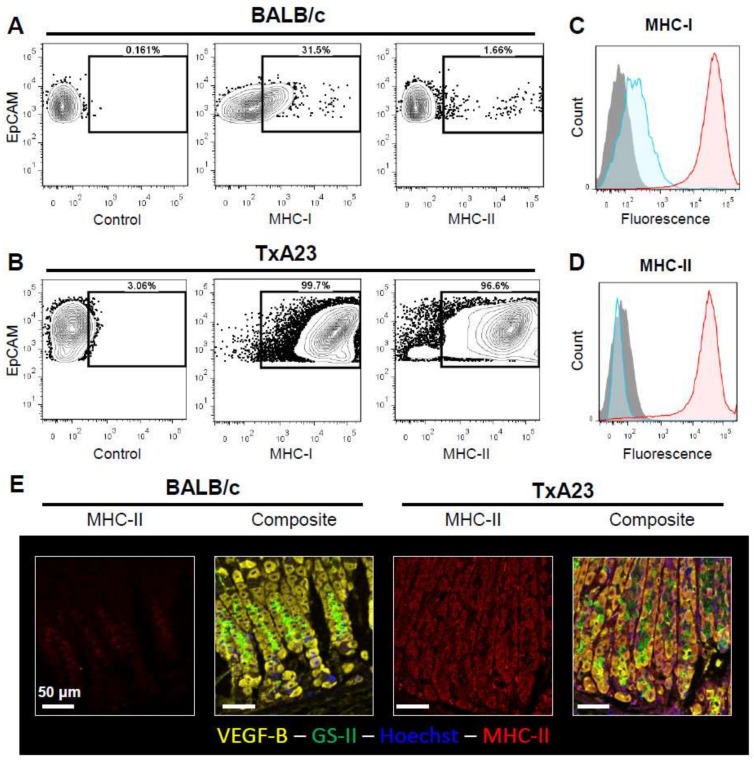
Gastric epithelial cells upregulate MHC-I and MHC-II in response to inflammation. (**A**,**B**) Representative flow plots of EpCAM+CD45-gated cells stained with antibodies to detect MHC-I or MHC-II surface proteins. Identical gating strategies show that MHC-I and MHC-II molecules are expressed at higher levels on gastric epithelial cells from mice with autoimmune gastritis (TxA23 mice). (**C**,**D**) Histograms showing the relative expression of MHC I and MHC II in BALB/c (blue) and TxA23 (red) mice. Data are representative of 2 experiments, 5 mice per group. (**E**) Representative images of gastric tissue sections from BALB/c and TxA23 mice stained with Hoechst (blue), VEGF-B (yellow), GS-II (green), and MHC-II (red).

Cell lines and immunofluorescent staining of gastric mucosal tissue sections have been used to demonstrate that gastric epithelial cells respond to inflammatory stimuli such as interferon-γ by expressing MHC-II [18,19]. Preparation of single cell suspensions involves the use of proteases that could alter the detection of proteins expressed on the cell surface by flow cytometry. To determine if our methodology was capable of detecting MHC-I and MHC-II upregulation directly ex vivo from chronically inflamed mucosa we analyzed MHC-I and MHC-II on gastric epithelial cells (GECs) isolated from normal BALB/c stomachs and chronically inflamed TxA23 stomachs. Analysis revealed that MHC-I proteins were upregulated >150-fold on TxA23 GECs compared to BALB/c (mean fluorescence intensity: 260 ± 19.5 vs. 42,390 ± 1783). Furthermore, while MHC-II expression levels were undetectable on BALB/c GECs, expression levels were >1500 fold higher on GECs isolated from mice with autoimmune gastritis (mean fluorescence intensity: 12.6 ± 12.6 vs. 19,990 ± 1682) (Figure 3A–D). Immunofluorescent staining of corpus sections from BALB/c and TxA23 confirmed that MHC-II protein expression was much higher on epithelial cells in the chronically inflamed stomach (Figure 3E). Overall these studies indicate that this method is both sensitive and specific to inflammation-induced changes in the gastric epithelium and can be used for direct ex vivo analysis of gastric epithelial samples.

## 3. Discussion

Microscopic analysis is standard in studies of the pathophysiology of gastric diseases. It allows for the qualitative assessment of disease phenotype and epithelial cell changes and, when coupled with techniques like immunofluorescence and immunohistochemistry, it also allows for comparisons of protein expression. However, quantitation of these data is time-consuming and highly dependent upon proper tissue preparation and sampling of many portions of the tissue in the case of focal disease processes. To assess gastric epithelial cell changes in our model of inflammation-induced gastric atrophy, TxA23, we have developed a reliable method for processing tissue and analyzing via flow cytometry that yields repeatable results.

Our method of tissue processing involves a two-step enzymatic digestion: first with collagenase to release the glands from the stromal tissue, followed by a digestion step with Dispase II that further separates glands into single cells. While previous publications have noted that enzyme digestion is harsh on cells and significantly effects viability, our use of this method generates an average of 1 million viable single cells per stomach (Figure 1C) [5]. Moreover, this digestion protocol requires no special equipment beyond a microtiter plate shaker placed at 37 degrees Celsius. We have also observed that gastric epithelial cells are very sensitive to centrifugation, with speeds above 50× gravity decreasing viability. This change in protocol could explain our improved cell yields despite the use of enzyme digestion. While previous studies have used various forms of enzymatic digestion, these publications reported additional steps involving mechanical disruption, microdissection, DNase incubation, or frequent media changes during digestion. However, we did not determine these additional steps to be necessary for successful isolation of viable single cells. [5,11,13] (Table 1).

Following single cell suspension, cells were stained with 7-AAD, a DNA-binding viability dye that efficiently discriminates between live and dead cells during flow cytometric analysis. Attempts to use fixable viability dyes that bind to free amines as a measure of cell viability proved difficult to interpret due to high background staining. This is possibly due to nonspecific staining of mucins, as 7-AAD binds to nucleic acid rather than protein substrates. Anti-EpCAM was also used to select only the epithelial cells, which is critical for distinguishing between immune and epithelial cells in models with significant inflammatory infiltrate. All staining was done in PBS supplemented with 0.5% bovine serum albumin and 2 mM ethylenediamineteraacetic acid (EDTA) to prevent aggregation of cells during staining. When analyzing, we first exclude cells by size and internal complexity using forward and side scatter of incident light. As GECs are much larger and more complex than typical lymphocytes, voltage levels for the forward-scatter and side-scatter detectors were adjusted accordingly. We then gated on viability dye negative, EpCAM positive cells for the remainder of the analysis. To verify the ability of this method to detect differential expression of surface markers, we stained epithelial cells with anti-major histocompatibility complex class I (MHC-I), which is present on most nucleated cells and compared it to the expression of MHC class II, which is a molecule typically limited to antigen presenting cells of the immune system. In normal BALB/c mice, we see typical expression of MHC-I and little to no expression of MHC-II on gastric epithelial cells. While antibodies against lineage markers of the gastric epithelium have been published as effective in differentiating the different cell types using flow cytometry [5,12,20], we found no staining conditions that were sufficiently specific to confidently distinguish between lineages using the published antibodies. However, we were able to identify parietal cells using the fluorescently conjugated DBA lectin that has been published in tissue staining as a parietal cell specific marker [16,17]. This is the first report of using flow cytometry to identify parietal cells according to staining with DBA. It should also be noted that use of a hematopoietic immune cell marker such as CD45 also allows for the analysis of infiltrating immune cells without interference from the more problematic epithelial cell populations, which has also been a focus of some groups [21]. We repeated these analyses using C57Bl6 mice, which are commonly used in mouse models of gastric cancer, and observed no difference in the efficacy of these cell isolation and FACs analysis methods.

It has been described that epithelial cells of the gastrointestinal tract, such as those of the small intestine, react to inflammatory stimuli by upregulating MHC-I and MHC-II and are even capable of presenting antigens to CD4+ T cells. It has also been shown that gastric epithelial cell lines exhibit this phenomenon and that MHC-II plays a role in the adherence of *Helicobacter pylori* to the gastric epithelium and induction of apoptosis by crosslinking of MHC-II molecules [18,19]. We wanted to use our method to perform a direct ex vivo analysis of inflammation-induced changes in MHC expression. To do this, we analyzed MHC-I and MHC-II expression on gastric epithelial cells of mice with chronic atrophic gastritis (TxA23) compared to normal gastric epithelium (BALB/c). We observed a significant upregulation in both MHC-I and MHC-II, which duplicates results seen by other groups using immunofluorescent staining. While we did not observe the expression of costimulatory molecules required for naïve T cell activation, MHC-II expression could implicate a role for gastric epithelial cells in reactivating infiltrating CD4 T cells during chronic inflammatory states, furthering disease progression by stimulating the production of more inflammatory cytokines. However, this requires further experimentation to determine unequivocally.

From these experiments we conclude that enzymatic digestion to single cells followed by flow cytometric analysis is an effective way to analyze large numbers of viable gastric epithelial cells, and that this method is useful for studying inflammatory changes in surface markers on gastric epithelial cells during chronic disease processes such as *Helicobacter* infection or autoimmune gastritis. It is anticipated that this method will enable valuable future studies in the gastric cancer field such as: analysis of immune activating or inhibitory receptors on gastric epithelial cells, changes in the expression of these receptors during inflammation, fluorescence activated cell sorting of gastric epithelial cell populations based on protein expression, and single cell RNA-SEQ analysis of gastric epithelium.

## 4. Methods

### 4.1. Mice

TxA23 mice express a transgenic T cell receptor specific for a peptide from H^+^/K^+^ ATPase alpha chain and spontaneously develop preneoplastic lesions such as parietal cell atrophy, mucous neck cell hyperplasia, and spasmolytic polypeptide expressing metaplasia [14,15,22,23]. BALB/c mice were purchased from Jackson Laboratories. All mice were maintained in our animal facility and cared for in accordance with institutional guidelines (Protocol 2600, Approved 6-07-2016 by the Saint Louis University Institutional Care and Use Committee).

### 4.2. Immunofluorescence/Immunohistochemistry

Stomachs were prepared, stained, and imaged using methods modified from Ramsey et al. [24]. The primary antibodies used for immunostaining were goat anti-VEGF-B (1:100 from Santa Cruz Biotechnology, Santa Cruz, CA, USA, sc-13083) and rat anti-mouse MHC-II (1:100 from BD Biosciences, San Jose, CA, USA, 556999). Secondary antibodies, nuclear labeling, and GS-II lectin labeling were as described.

### 4.3. Generation of Gastric Epithelial Single Cell Suspensions

Gastric glands were isolated from the corpus region of BALB/c and TxA23 mouse stomachs using collagenase (10 mg/mL from Sigma-Aldrich, St. Louis, MO, USA, C9891) in a digestion media comprised of advanced MEM (Gibco, Gaithersburg, MD, USA, 12492-013), 20 mM HEPES, 0.2% BSA (Millipore, Burlington, MA, USA, 810033), penicillin-streptomycin (Sigma-Aldrich, P7081), and 50 µg/mL gentamycin (Sigma-Aldrich, G-1914). The whole gastric corpus was separated from the forestomach, antrum, and esophagus and agitated at 600 rpm in collagenase for 30 min at 37 °C. Following digestion, remaining gastric serosa and connective tissue were removed from the glands. Glands were pelleted by centrifugation at 50× *g* gravity for 10 min and washed twice using DMEM/F12 (Sigma-Aldrich, D6421) supplemented with penicillin-streptomycin, gentamycin, and 0.5 mM 1,4-Dithiothreitol (DTT) (Sigma-Aldrich, GE17-1318-01). Glands were then digested into single cells by agitating at 600 rpm using Dispase II (Sigma-Aldrich, D4693) for 90 min at 37 °C. Following Dispase digestion, single cells were washed twice using flow cytometry staining buffer supplemented with 20 mM EDTA (Promega, Madison, WI, USA, V4231) and counted using Trypan blue exclusion. For Cytospins, glands and cells were stained using the Hema 3 Stat Pack (Fisher Scientific, Hampton, NH, USA, 123-869) and spun onto microscopy slides using the Cytospin 4 Cytocentrifuge (ThermoFisher, Waltham, MA, USA, A78300003). For immunocytochemistry, Cytospin preparations were fixed in 4% PFA for 10 min followed by permeabilization in 0.5% BSA/0.1% Triton X-100/ 2 mM EDTA and then stained according to immunofluorescent protocols listed above.

### 4.4. Quantitative Real Time PCR

Total RNA was prepared using RNeasy Mini Kit (Qiagen, Hilden, Germany, 74104). The quantity and quality of RNA was determined using a NanoDrop 2000 spectrophotometer (Thermo Scientific). cDNA copy of RNA isolated from cells was done according to the manufacturer’s instruction (High Capacity cDNA Reverse Transcription Kit, Applied Biosystems, Foster City, CA, USA). Quantitative PCR was run on the 7500 Real-Time PCR System (Applied Biosystems). The following primer/probe sets were used: *Gapdh* (Mm99999915_g1), *Atp4a* (Mm00444417_m1), *Gif* (Mm00433596_m1)*, Muc5ac* (Mm01276718_M1), *Muc6* (Mm00725165_m1).

### 4.5. Flow Cytometry

Cell surface staining was performed on gastric epithelial cells in staining buffer (PBS + 2% BSA) supplemented with 20 mM EDTA. Cells were kept on ice at all points during the staining procedure and analysis to prevent cell aggregation. Staining against surface antigens was performed using antibodies against Pan CD45 (BD Biosciences, San Jose, CA, USA 559864), EpCAM (eBioscience, San Diego, CA, USA, 47-5791-80), CD45.1 (BD Pharmigen, San Jose, CA, USA, 553776), MHC-I (BioLegend, San Diego, CA, USA, 125506), MHC-II (BD Biosciences, 557000). Following surface stain, cells were washed twice, passed through a 40 µm filter, and resuspended in staining buffer with 1 µL/mL 7-AAD (eBioscience, 00-6993-42) for dead cell exclusion. For identification of parietal cells, single cell suspensions were fixed in 4% PFA for 5 min at 37 degrees Celsius followed by permeabilization in 0.5% BSA/0.1% Triton X-100/2 mM EDTA for 30 min in the dark at room temperature on a plate shaker. *Dolichos biflorus* agglutinin conjugated to FITC (EYLabs, San Mateo, CA, USA, F-1201) was then added to cells at 1:500 and incubated for 1 h in the dark at room temperature. All flow cytometry was performed on a BD LSRII and analyzed using FlowJo (FlowJo, LLC, Ashland, OH, USA).

### 4.6. Statistical Analysis

Data are expressed as means of individual determinations ± standard error. Statistical analysis was performed by either the Mann-Whitney *U* Test, an unpaired Student’s *t*-test, or a two-way ANOVA with Bonferroni post-tests (* *p* < 0.05; ** *p* < 0.01; *** *p* < 0.001) using GraphPad Prism 5 (GraphPad Software, La Jolla, USA).

## Figures and Tables

**Table 1:** Comparison of Methods for Generating Single Cell Suspensions.

Reference	Disruption	Enzymes	Region	FACs Gating Description
Zavros et al.; 2000 [5]	Medimachine	Collagenase	Whole Stomach	No
Moore et al.; 2015 [11]	Medimachine	Dispase II	Corpus	No
Hinkle et al.; 2003 [13]	None	Pronase	Corpus	No
Bockerstett et al.; 2018	None	Collagenase + Dispase II	Corpus	Yes
